# The Blossoming of Technology for the Analysis of Complex Aroma Bouquets—A Review on Flavour and Odorant Multidimensional and Comprehensive Gas Chromatography Applications

**DOI:** 10.3390/molecules24112080

**Published:** 2019-05-31

**Authors:** Michelle S.S. Amaral, Philip J. Marriott

**Affiliations:** Australian Centre for Research on Separation Science, School of Chemistry, Monash University, Wellington Road, Clayton, VIC 3800, Australia; Michelle.Amaral@monash.edu

**Keywords:** MDGC, GC×GC, scents, fragrances, mass spectrometry

## Abstract

Multidimensional approaches in gas chromatography have been established as potent tools to (almost) attain fully resolved analyses. Flavours and odours are important application fields for these techniques since they include complex matrices, and are of interest for both scientific study and to consumers. This article is a review of the main research studies in the above theme, discussing the achievements and challenges that demonstrate a maturing of analytical separation technology.

## 1. Introduction

Multidimensional (MDGC) and comprehensive two-dimensional gas chromatography (GC×GC) are hyphenated separation techniques, which address separation difficulties faced in complex sample analysis that require superior resolution in order to achieve improved analysis of compounds. Both techniques can also be applied to achieve faster separations in less complex samples, especially when there is an important region to be better resolved. Relatively few publications report the parallel analysis of samples by both a GC×GC and MDGC method, compared with a carefully optimised 1D GC method. In some cases, commentary is made that the MDGC method permitted identification or separation of many more compounds, and so this is accepted as stated. On a simple capacity basis, GC×GC and MDGC outperform 1D GC, although it is recognised that many 1D GC methods are not applied to give an ultimate resolution performance. While MDGC is composed of two (or more) separation steps that progressively ‘deconvolute’ target analytes or a fraction, GC×GC is a two-dimensional modulated untargeted separation, which is used to obtain improved resolution of the whole sample. The separation columns are referred to as dimensions—first column or dimension ^1^D and second column ^2^D—which highlights that they operate as independent elution stages. Both techniques use substantively different stationary phases in each dimension, in order to obtain improved resolution by means of changing the separation mechanism (orthogonality). Although multidimensional GC separations have been applied since the early 1960s and the comprehensive technique was introduced in the early 1990s, the number of publications in this field was boosted in the past 20 years by the continuous technological advances in instrumentation, software and data processing [1,2,3]. 

Among the main applications to which MDGC and GC×GC have been extensively applied is flavours and fragrances analysis [2]. The study of these matrices, as well as their authentication and quality control is indispensable, since it is well known that the smell and taste are important quality markers which boost the consumers’ choice of food, drinks and beverages, fragrances, cosmetics and other products [4,5]. Furthermore, aromatherapy is another important emerging application of odorous products, which is directly connected to human behaviour and health, areas that are gaining prominence in aroma science [5].

Sample preparation techniques are often used to increase analyte concentration. Likewise, MDGC can increase concentration through making multiple injections, whilst maintaining resolution. This increase in sensitivity corresponds to greater chemical and sensorial responses. The complexity of many matrices, including a multitude of (semi-)volatile compounds, from different chemical classes and ranging from major to trace concentration, makes it harder to achieve reliable identification and quantification in a single dimension separation. In addition, important (odour/flavour-active) stereoisomers are usually present, which may lead to ambiguous mass spectrometry library matches [2,5,6]. An interpretation of usefulness, advantages and limitations of MDGC and GC×GC techniques are compared in Table 1.

The analyst may rely on different chromatographic systems and detectors to achieve improved sample characterisation. This is usually performed by matching the chromatographic information (e.g., retention times, retention indices) of sample analytes and authentic standards with the response of different detectors. The detectors can be general/universal (e.g., flame ionisation detection—FID, mass spectrometry—MS, Fourier transform infrared—FTIR, vacuum ultra-violet—VUV), element-selective (e.g., flame photometric detection—FPD, nitrogen-phosphorus detection—NPD, electron capture detection—ECD) or sensorial (e.g., olfactory port—O, electronic nose—e-nose, electronic tongue—e-tongue) [7]. Important detectors for determination of aroma-active compounds are the mass spectrometer and the olfactory port, which provide a measure of chemical and sensorial identification, respectively. The continuous development and commercial introduction of new stationary phases, such as ionic liquids and cyclodextrins, may address new mechanisms of separation, but in complex samples may do little to expand the separation capacity in 1D GC. Thus, the problem of incomplete separation of components remains, and for the MS and ‘O’ detectors, generally complete resolution provides best affirmation of chemical properties or identification. 

**Figure 1:**
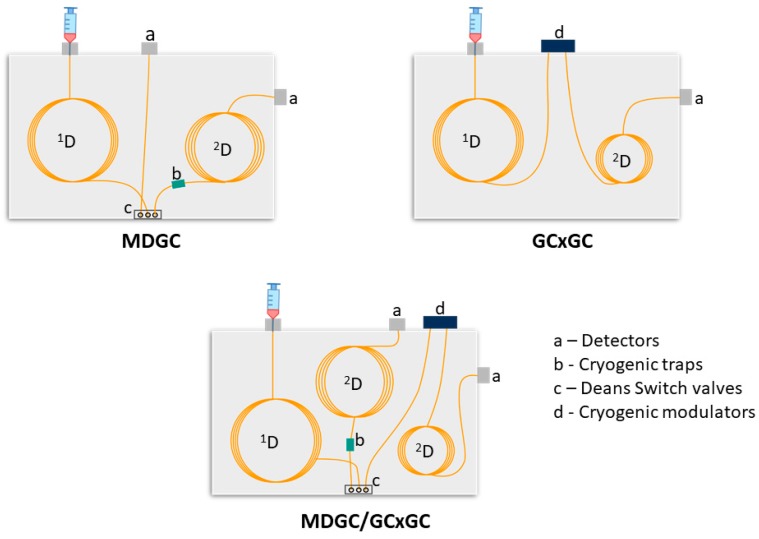
Simplified schematic examples of different multidimensional systems: MDGC, GC×GC and integrated MDGC/GC×GC.

Different design configurations are continually being explored for multidimensional GC techniques (Figure 1), using extended or integrated MDGC and GC×GC systems [8,9,10,11,12,13,14,15,16], coupled with different detectors, to address even more demanding separations and to accomplish greater separation. The overriding aim is to expand the separation capacity of the system. MDGC (Figure 1) does this by taking heart-cuts of specific regions on a first column and passes them to a conventional length second column for greater separation, which is of particular value in enantioselective analysis where the ^2^D column comprises the enantioselective phase. A cryotrap assists in reducing ^1^D dispersion. By contrast, GC×GC (Figure 1) uses a modulator device (Figure 1) in conjunction with a short ^2^D column to rapidly and sequentially transfer solute between the two columns, according to the modulation period, to produce a significantly increased overall capacity for the total sample. Presentation is conveniently expressed as a 2D plot—with axes ^1^D and ^2^D time—with individual compounds located throughout the plot according to their chemical/physical properties and interaction with the stationary phases. A further option of hybrid nature (MDGC/GC×GC; Figure 1) allows operations such as select target zones, improve their separation, and subject them to GC×GC. 

Marriott and co-workers contributed a number of technical solutions and strategies to achieve better resolution of aroma compounds. Their successful implementation of a number of hybrid GC approaches in a single instrument [8,9,10,11,12] represents qualitative and quantitative developments to provide a convenient solution to overcome uncertainty of data correlation across multiple hyphenated systems. The use of cryogenic trapping and modulators, with flow switching explored by the group [17,18,19,20], allows the focussing and/or concentration of the volatile analytes in various modes of operation, which improves separation peak capacity and enhances the quality of analyte characterisation. 

Food, beverages and essential oils matrices may represent the top three applications when considering flavour and odour analysis. Beyond these obvious fields, odour analysis is also applied in other diverse areas such as environmental, biochemical and forensic sciences, as well as in analysis of wood, incense, tobacco and plastic materials. The sections below will concentrate on discussion of the main applications of MDGC (also called 2DGC in some works) and GC×GC techniques in flavour and odour analysis, according to type of sample, with future perspectives considered in the conclusion.

As a disclaimer, in the applications which follow, some studies claim to detect compounds that had not previously been reported in particular samples, attributed to new higher resolution separation technology. We reproduce these claims, but do not examine the veracity of these, nor subject them to scrutiny as to whether they have completed thorough processes in ‘discovery’ of these compounds such as retention indices on two different columns, comparison and high correlation of mass spectra, and use of authentic reference compounds. 

## 2. Food

Food aroma and flavour are formed by a complex mixture of volatile and non-volatile substances. Among this rich mixture, there are different active-compounds, which are responsible for the taste, odour, texture and other sensorial perceptions and, therefore, are the focus of many studies in food science. Comprehensive and multidimensional GC analysis are indispensable tools to identify, quantify and correlate the key compounds (volatiles and semi-volatiles) to their sensory activity, as well as performing a well resolved fingerprint or profile [5,6]. Among sample types investigated in this field with application of MDGC and GC×GC techniques include fruits, nuts, honey, truffles, oils, herbs and spices, meats and other products.

### 2.1. Fruits and Nuts

Fruits are widely traded commodities with attractive flavours, aromas, colours and a number of benefits to health, such as their contents of vitamins, antioxidants, anti-carcinogens and other active compounds. They are consumed raw, prepared as drinks and beverages, flavourings, perfumes, essences, cosmetics, medicines, etc. Revealing the composition of fruit aroma and flavour, the use of MDGC and GC×GC techniques resolve their complex profiles, and with enantioselective separation strategies the composition may be determined. Enantiomeric separation preferably demands complete resolution of the isomers from matrix to allow evaluation of impact and characteristic odour, and supports their use as authentication markers [21,22]. 

Mastello et al. [21] used MDGC-O/MS, GC×GC-FID and GC×GC-time-of-flight mass spectrometry (TOFMS) techniques to identify odour-active volatiles in processed orange juice, providing better resolution for seven most potent odour regions identified from 13 previously identified through GC-O. Confirmation by matching the identity and odour of four aldehydes (hexanal, heptanal, octanal, citral), two esters (ethyl butanoate, methyl hexanoate) and four monoterpenes (α-pinene, D-limonene, linalool, α-terpineol) was established.

Species of *Citrus* fruits, such as *C. hystrix* [22], *C. australasica* [23], *C. junos*, *C. limon* and *C. aurantifolia* [24], and the *Citrus*-like *Eustis limequat* fruit [25] were analysed by MDGC and GC×GC techniques which proved to be efficient tools to differentiate odorants, varieties and origins, as well as reliably separate, identify and quantify their chiral compounds. 

Lubinska et al. [22] analysed fresh peel and pulp of kaffir lime (*Citrus hystrix*) fruits by GC×GC-TOFMS to identify and quantify key aroma compounds (α-pinene, limonene, citronellal, linalool, terpinen-4-ol, myrcene, α-terpineol, and citral) and their bioactive properties for cosmetology applications. Peel and pulp had different volatile profiles. The major compounds identified were citronellal (peel) and terpinen-4-ol (pulp). Terpinene compounds, characterised by a woody-earthy odour, were present in higher amounts in the pulp of the fruit, while the citrus-fruity odorants dominated peel extract, and explains the perceived differences in the smell of both fruit parts.

Delort et al. [23] and Hong et al. [24] assessed enantiomeric ratios of the chiral aroma volatiles from Australian finger lime (*Citrus australasica*) and other three *Citrus* species (*C. junos*, *C. limon* and *C. aurantifolia*), respectively, using MDGC systems. In the former work, the authors identified six new terpenyl esters and some isomers in the finger lime samples: 6-methyloctyl acetate, citronellyl citronelate, 1,2:5,6-diepoxy-*p*-menthane, 2,3-epoxy-*p*-menthan-6-one, *cis-* and *trans*-*p*-menth-1-en-3-ol-6-one and 1,2-epoxy-*p*-methan-5-one, some of them reported for the first time in a natural product. Moreover, *cis*-isoascaridole was confirmed in the fruit [23]. The other three *Citrus* species assessed in the second study, had limonene, γ-terpinene and linalool as major compounds and excess ratios of the chiral compounds *R*-(+)-sabinene in *C. junos*, as well as more *S*-(−)-sabinene in *C. limon* and *C. aurantifolia*. Such information can be used as authenticity and quality indicators for the species analysed [24]. Casilli et al. [25] used a selectable MDGC-MS/O system to characterise, resolve complex regions, determine enantiomeric ratios, and assess the odour activity of the peel of *Eustis limequat* fresh fruits, allowing the identification of trace constituents, without fractionation and also enabled the confirmation of (*R*)-(−)-cis-*δ*-jasminlactone in the *Citrus*-like fruit.

**Figure 2:**
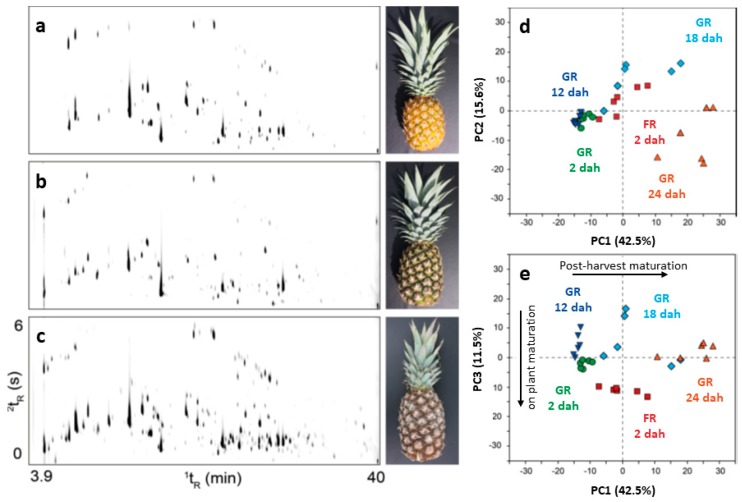
Pineapple volatile profiles (GC×GC-quadrupole MS (qMS)) at different ripening stages: (**a**) FR 2 dah; (**b**) GR 2 dah; and (**c**) GR 24 dah. Principal component analysis score plots (**d**) PC1 × PC2 and (**e**) PC1 × PC3. GR: green-ripe; FR: fully ripe; dah: days after harvest. PC1: postharvest maturation; PC2: late postharvest maturity stages of sea-freighted pineapples; PC3: plant ripened pineapples. Source: adapted with permission from Steingass et al. [27].

Tropical fruits, such as pineapples [26,27] and passion fruit [28,29] and their unique aromas were also investigated by GC×GC and MDGC techniques. Steingass et al. [26,27] assessed the aroma volatiles of green-ripe and fully ripe pineapples of different postharvest maturity stages (Figure 2), using GC×GC–MS, and submitted their results to principal component analysis which illustrated clustering of similar sample maturity. The higher sensitivity and resolution power reportedly allowed the identification of 291 compounds, from which 97 were described for the first time in the sample. The main classes identified were esters, terpenes, alcohols, aldehydes, ketones and lactones, and fatty acids and their esters. In their later study, more than 400 detected signals were submitted to principal component analysis. Differences in the volatile profiles were observed for the four ripening stages investigated (2, 12, 18 and 24 days after harvesting), of which 25 substances, mostly esters, were tentatively identified and suggested as maturation marker compounds. GC×GC allowed the discovery of postharvest genesis of mono and sesquiterpenes, which could be induced as a self-defence mechanism to fungal infection.

Yellow (*Passiflora edulis* f. *flavicarpa*) and purple (*Passiflora edulis* Sims) passion fruit flavours and odours were investigated by Werkhoff et al. [28] and Strohalm et al. [29], using different MDGC approaches. In the former work, the authors used the MDGC system to obtain an enriched fraction of sulfur compounds, and to determine the enantiomeric distribution ratios for 20 substances of the yellow passion fruit. Forty-seven sulfur-containing compounds were identified, of which 35 were found for the first time in the sample and 23 not been previously reported as flavour constituents in food, such as hexyl 3-(methylthio)propanoate (fruity/geranium-like odour). Furthermore, among the chiral substances evaluated, it was found that sulfur-containing volatiles with *S* configuration were present at the highest amount in passion fruit samples [28]. The enantioselective analysis of passion fruit aroma volatiles was also explored by the latter work, assessing the chiral ratios for secondary alcohols and their esters in both yellow and purple fruit types. The predominance of *R*-enantiomers for the 2-alkyl esters were observed for both fruits. In addition, an opposite configuration in each fruit type was observed for 2-heptanol and 2-pentanol, having higher amounts of the *S* configuration in the purple fruits and higher-to-racemic amounts of the *R* configuration in the yellow fruits [29].

Berry fruits are another important class with particular properties. Their aroma and flavour were the focus of various MDGC studies, including strawberries [30,31], raspberries [32], blue honeysuckle, chokeberry, bilberry [33]. Samykanno et al. [30], applied GC×GC-TOFMS to obtain the volatile profiles of two varieties of strawberries harvested in Australia (Albion and Juliette). They identified a total of 94 volatiles of which 20 were not previously reported in the literature. Cannon et al. [31] also analysed strawberry samples of Ciflorette variety, using a MDGC-MS system. According to the authors, the methodology allowed the identification of 252 compounds not previously reported in strawberries. In addition, 24 volatile sulfur compounds were found in the extracts, and resolution of otherwise co-eluting carboxylic acids was achieved. The aroma of the extracts was evaluated by panellists through a GC-O interface and a total of 43 odour-active compounds were identified. 

Cocoa and hazelnut odorants were assessed by Cordero and co-workers [34,35], using GC×GC and MDGC approaches. The techniques allowed the determination of the volatile fingerprints, identification of potent odorants and correlation of datasets to the product’s origin and/or processing chain. Differences in the amount of the compounds from each class can be observed in cocoa samples, according to the origin or processing step. For hazelnuts, the combination of optimal drying (18–20 °C) and storage (atmosphere with 99% N_2_/1% O_2_) conditions were found to be the crucial parameters to preserve the raw product aroma. Kief et al. [36,37] studied the influence of the processing on hazelnut aroma, applying GC×GC-TOFMS analysis to identify and quantify the main odorants released from raw and roasted samples from different cultivars. They found 24 key-aroma compounds in the analysed samples and observed a dose-response relationship between the concentration of six compounds with nutty/roasty notes and the degree of roasting, which is directly related to the quality of the product. 

### 2.2. Honey

As a popular natural food product with a distinctive flavour, honey was investigated by Siegmund et al. [38], who used GC×GC-MS to compare the volatiles and sensory profiles of Austrian and Mediterranean honeys. Eight different monovarietal/unifloral samples according to their source were studied: robinia, rapeseed, dandelion, fir tree, orange, lavender, linden and chestnut honeys. The GC×GC approach enabled separation of co-eluting ^1^D compounds and the identification of 34 compounds described for the first time in dandelion honey. The authors also identified odour-active compounds that were not related to the samples, suggesting that their origins were probably related to contamination with essential oils.

### 2.3. Dairy Products

Dairy foods are very common in the human diet; the aroma of some products have been investigated by applying GC×GC techniques [39,40]. Butter flavour compounds were analysed by GC×GC-FID and GC×GC-TOFMS, with superior resolution enabling detection and identification of many more compounds, compared to the 1D approach, along with more reliable quantification of some target analytes. The main classes identified were aldehydes, alcohols, enals, ketones, fatty acids, alkanes, lactones, furans, pyridines and pyrroles [39]. The lactones responsible for the milky odour in dairy products were the focus of another study, which used GC×GC-TOFMS to quantify some γ- and δ-lactones in raw and pasteurised cream samples. This demonstrated that heating increased the concentration, mainly, of the *δ*-lactones and therefore the process could be a way to change the aroma of the products [40].

### 2.4. Flour and Pasta

A MDGC method was used by Costa et al. [41] to obtain aroma volatile profiles of two types of flour and related pasta products. GC parameters, especially the automatic pressure control and flow rates were optimised to allow complete transfer of the analytes from ^1^D to ^2^D. A multi Deans switch was used and the repeatability of the ^1^D retention times were evaluated. An automated multiple headspace SPME sampling method was developed and optimised for quantification purposes. The technique allowed correlation between products (raw and processed), by identification of substances in common in the samples. Chiral separation enabled identification of 14 enantiomer pairs and their ratios in the samples, of use to evaluate authenticity of the ingredients. 

### 2.5. Meats and Seafood

As a popular source of protein in human diet, accompanied by an appetizing aroma, several works have explored multidimensional techniques for meats and seafood, investigating beef [42,43], lamb [44], hams [45,46], shrimp [47], seabass [48] and eel [49].

Chaintreau and co-workers [42,43] used GC×GC-TOFMS and olfactometric analysis to assess roasted beef sulfur impact odorants. Seven key substances were confirmed in the top note of the sample’s aroma, reported for the first time as beef odorants.

Thomas et al. [45] and Wang et al. [46] developed GC×GC studies of ham aroma volatiles to identify sulfur odorants in a cooked sample, and obtain volatile profiles to differentiate three dry-cured samples, respectively. Odorants present in trace amounts in cooked hams included 2-methyl-3-furanthiol, 2-methyl-3-(methyldithio)furan and bis(2-methyl-3-furyl) disulfide [45]. Increased resolution power enabled separation and identification of dihydro-4-hydroxy-2(3H)-furanone and acetic acid (co-eluting in 1D GC), as well as another six differentiating odorants in dry-cured Chinese hams [46].

Fresh and grilled eel samples were analysed by Huang et al. [49], using GC×GC. The authors identified around 39 more compounds with this technique than with 1D GC. The volatile fingerprint for both fresh and grilled samples was clearly different. The main odorants suggested for the grilled eel were methyl propyl disulfide, dimethyl trisulfide, heptane, octane and camphene. The results were converted into “odour barcodes,” based on the odour activity values, concentration ratio and odour descriptions.

### 2.6. Seasonings and Related Samples

Herbs and spices, edible oils and other cooking ingredients are a source of flavour for food preparation and/or consumption. GC×GC and MDGC were applied to the analysis of these products, including coriander [50,51,52], curry leaves [53], dried fennel seeds [54], ginger [55], virgin olive oil [56], rapeseed oil [57,58], vinegar [59], tomato-onion puree [60] and truffles [61,62].

**Figure 3:**
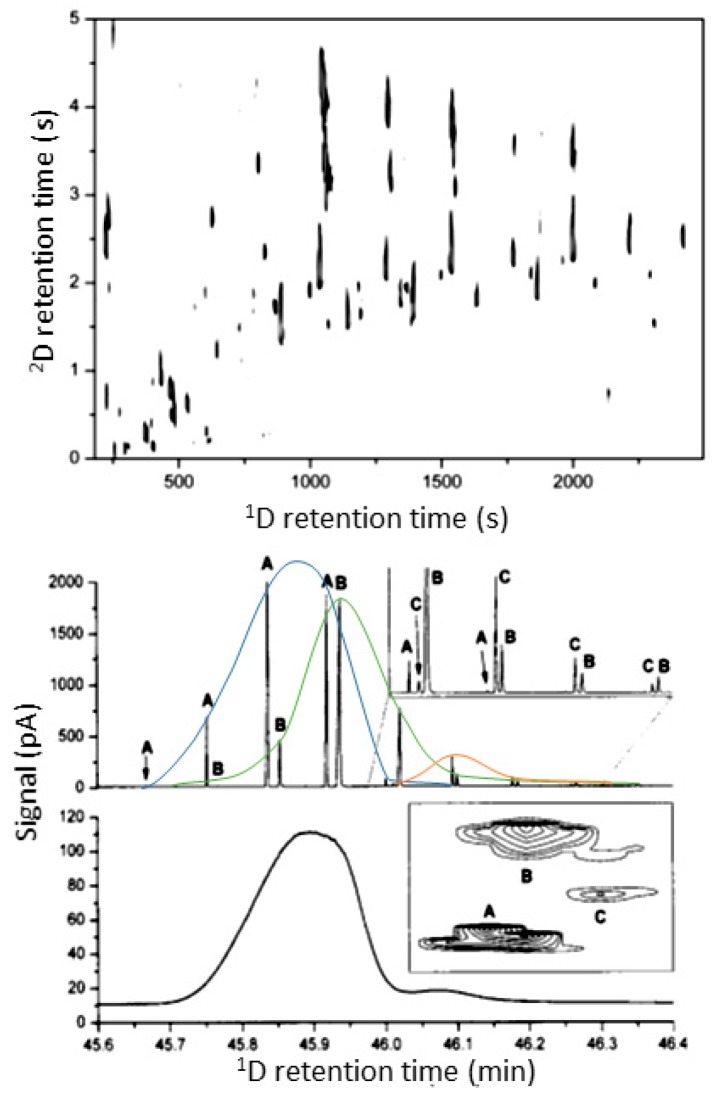
GC×GC-TOFMS volatile profiles of coriander (**top**). Separation of a potent floral, coriander-like odour cluster in coriander leaf (**bottom**). Substances were identified as *E*-2-dodecanal (A), *E*-2-dodecen-1-ol (B) and 1-dodecanol (C). Source: adapted with permission from Eyres et al. [50,51].

Coriander (*Coriandrum sativum*) and wild coriander (*Eryngium foetidum*) were the focus of Eyres et al. [50,51,52], in which the volatile profiles were identified for character impact odorants by using GC×GC-TOFMS, MDGC and GC-O analysis. The comprehensive technique allowed the identification of more than 50 compounds in both samples with a number found for the first time, reproduced in Figure 3. Alcohols, aldehydes, hydrocarbons and terpenes were the main classes present. A clear delineation of homologous compound series in the 2D GC×GC space was an advantage recognised by the authors, making isomer identification easier due to the relationship of related peaks in the 2D plot. The most important odorants found in *C. sativum* were (*Z*)-2-decenal, *β*-ionone, eugenol and the co-eluting cluster (*E*)-2-dodecenal, (*E*)-2-dodecen-l-ol, and 1-dodecanol. In *E. foetidum* the main odour compounds were (*E*)-2-dodecenal, eugenol, β-ionone and (*Z*)-4-dodecenal. 

Curry leaf aroma compounds and odour activity values were assessed by Steinhaus [53], using a MDGC system. The study confirmed that 1-phenylethane-1-thiol (sulfury/burnt notes) is the main impacting aroma compound present in the leaves and that the formation of other odorants, such as (3*Z*)-hex-3-enal, (3*Z*)-hex-3-en-1-ol (grassy notes) and (2*E*,6*Z*)-nona-2,6-dienal (citric notes) can be increased by enzymatic processes unleashed after the leaf tissue was disrupted.

Dried fennel seeds (*Foeniculum vulgaredulce*) were analysed by Maikhunthod and Marriott [54] using an integrated GC-O/GC×GC-FID and a GC×GC-TOFMS system to assess aroma-impact compounds. The main odorants identified were limonene, 1,8-cineole, fenchone, camphor, estragole, *trans*-anethole and *p*-anisaldehye. The authors reported a relationship between higher amounts of terpenes and freshness of the sample, evaluated using different shelf-aged fennel seeds. This characteristic can be observed in GC×GC profile results, and represents a facile approach to assess the freshness and, therefore the quality of the sample.

Sghaier et al. [57] developed a GC×GC method to resolve and quantify the main off-flavour compounds (1-penten-3-one, 1-octen-3-one, (*Z*)-4-heptenal, (*E,Z*)-2,6-nonadienal, (*E,Z*)-2,4-heptadienal, and (*E,Z,Z*)-2,4,7-decatrienal) responsible for the fishy-odour in rapeseed oil after thermal treatment. This allowed a two-fold increase in the number of identified compounds compared to classical one-dimensional analysis, based on improved separation.

Zhenjiang aromatic vinegar was analysed by Zhou et al. [59], who tentatively identified 360 compounds with the GC×GC-TOFMS technique and assessed the product’s odour activity by GC-O. The most potent odorants identified were acetic acid, 2-methyl-butanal, 3-methyl-butanal, dimethyl trisulfide, trimethyl-pyrazine, furfural, phenethyl acetate, 3-methyl-butanoic acid, 2-methyl-butanoic acid, 2-methyl-propanal, octanal, 1-octen-3-one, 3-(methylthio)-propanal, benzeneacetaldehyde and methanethiol. According to the authors, the latter seven compounds were reported for the first time as key odorants in the sample.

Sciarrone et al. [61] used a MDGC system coupled to a combustion-isotope ratio mass spectrometer (MDGC-C-IRMS) to assess authentication of truffle (*Tuber magnatum* Pico) and related products, monitoring the key-aroma compound bis(methylthio)-methane. As truffle is one of the most expensive food flavouring products, and its main aroma component can be easily and legally added to commercial products, the authors employed IRMS to determine the ^13^C/^12^C ratio abundance of the natural and synthetic analyte to track product authenticity. The authors reported a range of −33.9% to −42.6% for δ^13^C for bis(methylthio)-methane in 24 genuine truffle samples analysed, while the natural-origin and petrochemical standards had values of −28.5% and −56.4% or lower, respectively. Among the 14 truffle-based products analysed they found only eight with compatible δ^13^C values that confirmed a natural source. However, two of these were declared to be from black and summer truffle, which do not contain the evaluated analyte that was probably derived from a natural additive. All the remaining samples had δ^13^C values below −50%, indicating the addition of a synthetic flavour.

Two different Chinese truffle samples (black and white) were also analysed by Zhang et al. [62], using GC×GC-TOFMS. The profile of the both samples was dominated by acids, alcohols, phenols, aldehydes and esters. However, the samples can be distinguished by some differences observed in composition, mainly by the higher concentration of sulfur compounds in the white truffles.

## 3. Drinks and Beverages

Whether it is to refresh, stimulate, calm, nourish, quench thirst or just for socialising, drinks and beverages are consumed for a multitude of purposes. With a huge and diverse worldwide market, innovation and improvements in these products are driven by perceived consumer preferences and demand. Flavours and odours are among the main quality markers of these products—such as natural flavoured water products—usually composed of complex flavour extracts. Thus, several works applied GC×GC and MDGC techniques to assess the composition of non-alcoholic drinks (e.g., teas [63,64,65,66,67] and coffee [9,68,69,70,71,72,73]), as well as alcoholic beverages (e.g., wine [8,9,15,69,74,75,76,77,78,79], brandy [74], beer [68,80,81,82] and hop [83,84,85], cider [86], vodka [87], whiskey [14,74], cachaça [88] and spirits [74,89]). 

Qian and co-workers [63,64] assessed the flavour stability of iced tea products through MDGC, under different storage conditions. Although no new compounds were identified after the sample storage, they observed a decrease in concentration of many terpenes and some inversions in the enantiomeric configuration for compounds, such as (*R*)-(−)-linalool to (*S*)-(+)-linalool and (*S*)-(−)-*α*-terpineol to (*R*)-(+)-*α*-terpineol, which can be an explanation for the sensorial differences perceived between the fresh and aged samples.

Brazilian Arabica roasted coffee aroma was investigated by Miyazato et al. [72,73], who used MDGC-MS and GC-MS/O to determine odour-active trace compounds, as well as the off-odorants responsible for sweaty smell and a possible metabolic pathway for their formation. The authors reported having found the compounds 5-vinylguaiacol (phenolic, banana-like, sweet), 3-phenylpropionic acid (honey-like), *cis*-2,6-dimethyl-1,4-cyclohexanedione (pungent, warming, musty, mouldy), (*E*)-4-methyl-3-hexenoic acid and its (*Z*) isomer (sweaty), for the first time in the sample. The cyclic diketone was formed through thermal degradation of monosaccharides in alkaline conditions. The last two compounds may be derived from L-isoleucine and sugars, via Maillard reaction. 

**Figure 4:**
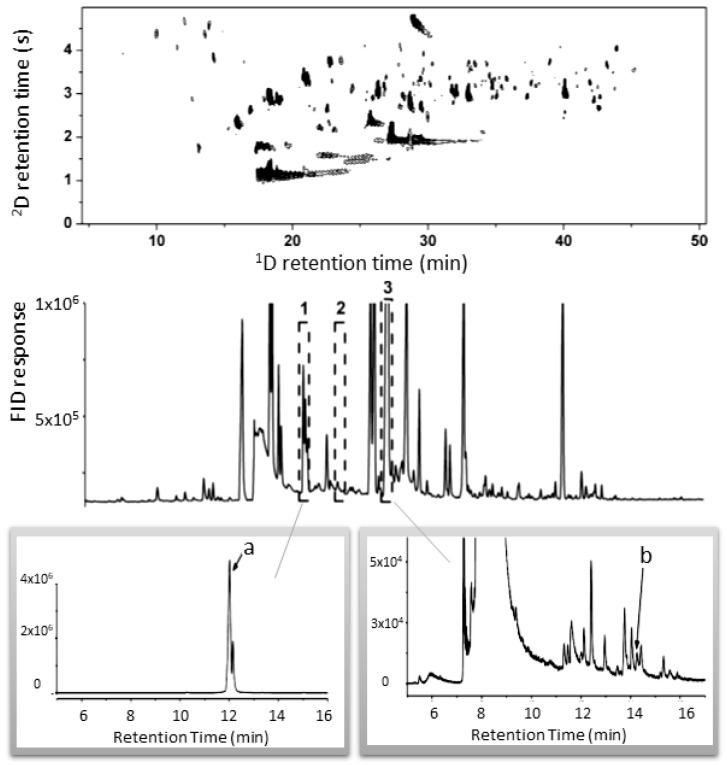
GC×GC- flame ionisation detection (FID) volatile profile of ground roasted coffee (**top** panel). ^1^D GC-FID chromatogram indicating three regions to be heart-cut (**centre**). ^2^D GC-FID results of the heart-cut of regions 1 and 3 (**bottom**). Peaks (a) and (b) are odour-active compounds responsible for the nutty and floral notes perceived in the target regions, respectively. Source: adapted with permission from Chin et al. [9].

The Marriott research group [8,9,69,88,89] studied the aroma composition of coffee (Figure 4) and different alcoholic beverages, such as wine, spirits and cachaça, applying different GC×GC and MDGC approaches, including integrated systems permitting MDGC and GC×GC and olfactometry in the same system (see Figure 1). Heart-cutting strongly overlapped regions from the ^1^D column to a high resolution ^2^D column allowed easier correlation of olfactometry and MS data. Banana Terra spirits were investigated for the first time by the authors, who identified 18 aroma-impact regions, and the main odorants found were 3-methylbutan-1-ol, 3-methylbutan-1-ol acetate, 2-phenylethylacetate and phenylethyl alcohol [89]. The same group also studied hop essential oil [12,52,83,84], identifying some of its impact odorants, such floral notes such as geraniol, linalool, *β*-ionone, and eugenol, and a compound responsible for woody/spicy aroma, 4-hydroxy-*β*-caryophyllene. 

Ochiai and co-workers [14,15,68,82] studied coffee and alcoholic beverages, such as beer, wine and whiskey, applying GC×GC and a selectable one-/two-D system. Detection of substances in very low levels (ng and pg) were achieved in the analysis of all samples with the selectable ^1^D/^2^D system coupled with element specific and chemiluminescence detectors. The aroma profile of beer was obtained by Stefanto et al. [82], using GC×GC-TOFMS and multiple statistical analysis to assess the most representative flavour compounds. 

Campo et al. [74] developed a MDGC method to identify and quantify four impact esters odorants in wine, brandy and whiskey samples. The method enabled assessment of the levels and olfactory impact of these compounds in different beverages, which may be related to the sweet fruit notes in some samples and can be formed by a slow esterification during the aging process of wines. 

## 4. Essential Oils and Fragrances

A precious mixture of secondary metabolites, mainly composed of mono- and sesqui-terpenoids and phenyl propanoids, which can be extracted from several plant parts, such as flowers, fruits, seeds, leaves, buds, roots and bark, the essential oils have remarkable economic value and applications ranging from flavours and fragrances to cosmetics and pharmaceutics [90]. 

Having many analytes from similar classes and with chiral properties, essential oils and related compounds and products are widely explored by MDGC [18,20,25,52,91,92,93,94,95,96,97,98,99,100,101,102,103,104,105,106,107,108,109,110] where the most appropriate orientation for chiral analysis is to use an enantioselective (*e*) ^2^D column (GC-*e*GC) [111]. The basic purposes include to obtain qualitative profiling and fingerprinting, identify and quantify target analytes, and assess odorants and allergens [104,105,112,113,114]. There are many aspects that can influence the chemical profile of these oils, such as species, chemotype, plant origin and age, environmental conditions, extraction method and processing (e.g., plant drying, oil deterpenation) [115]. Notwithstanding, different degrees of complexity can be expected for perfumes and fragranced products, depending on the formulation, which can comprise natural and synthetic aroma substances of several classes, with different odour activities and concentrations [91,105]. 

The range of component polarities in these samples, allows use of either a less polar ^1^D/more polar ^2^D set or more polar/less polar set for GC×GC analysis. For enantioseparation, in contrast to MDGC above, the chiral column should be the ^1^D column, i.e., *e*GC×GC [111,112,115].

Fidelis et al. [97] studied the essential oil obtained from the leaves of rosewood (*Aniba rosaeodora* Ducke), from plants of different ages, as a potential, more sustainable, alternative to extraction from the chipped wood. The chemical profile of the leaf oil samples from plants of different ages was similar and was examined by GC×GC, enabling a three-fold increase in the number of compounds reported than a conventional separation. 

**Figure 5:**
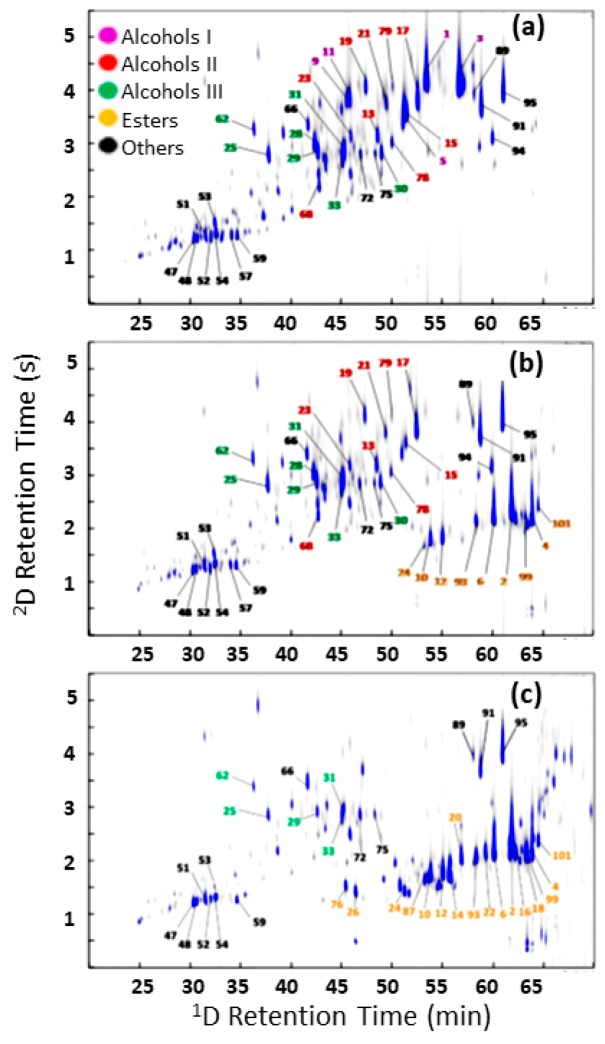
GC×GC-mass spectrometry chromatograms of (**a**) the natural Haitian vetiver essential oil, (**b**) the enzymatically acetylated oil, and (**c**) chemically transformed into vetiveryl acetate. The compound identity can be consulted in the source literature: adapted with permission from Francesco et al. [101].

Filippi et al. [98] conducted a qualitative and quantitative study of vetiver essential oil components, using a GC×GC-FID/MS system. The method allowed the identification of 135 compounds in four different samples by dosing in internal calibration standards, as well as observation of differences in composition of each sample related to their origins and ages. Tissandié et al. [102] studied the composition of different samples of a perfume ingredient called “vetiveryl acetate,” an improved odorant product derived from vetiver essential oil processing. The GC×GC-MS/FID analysis allowed identification of more than 200 compounds in the samples, generating a new dataset that can be used to assess the impact of the manufacturing process on essential oil composition and odour properties. Francesco et al. [101] used GC×GC to assess the composition of natural vetiver oil with transformed products—a vetiveryl acetate biocatalysed product, obtained from a lipase-catalysed acetylation of vetiver essential oil. The esterification process was highly chemoselective towards primary sesquiterpene alcohols, and had more sustainable processing features (i.e., mild reaction conditions, reusable catalyst, etc.). The use of the comprehensive technique was indispensable to deeply understand and compare and contrast the differences of the crude and the modified oils (Figure 5), since the matrix is highly complex and not suitable for discovery purposes by using the poor resolution of a 1D GC method.

Flower stereoisomer odorants, linalool and lilac aldehydes/alcohols, from 15 different plant species were assessed by Dötterl et al. [92], using *e*-MDGC separations and electrophysiological tests in a noctuid moth. Eight lilac aldehydes and their alcohol isomers were identified, and found to induce the insect response. The *S*-configured isomers were predominant in almost all the plant species studied. The technique was indispensable as a route to identify and collect the individual stereoisomers.

**Figure 6:**
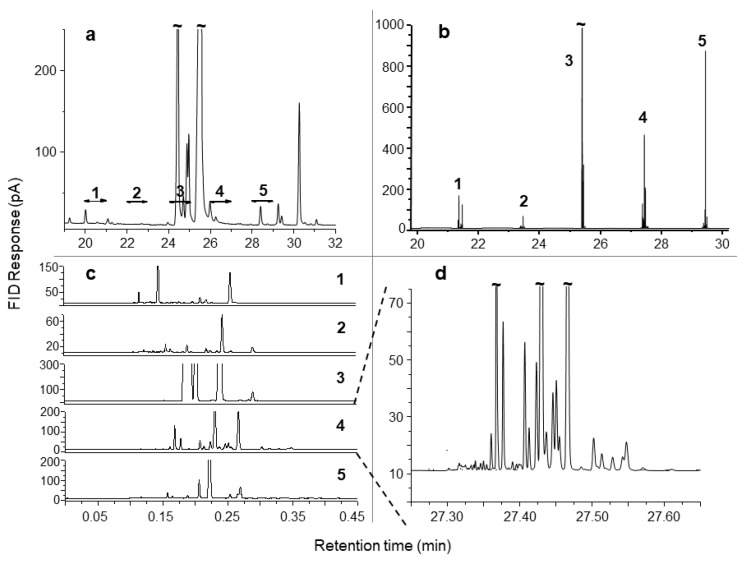
Peppermint essential oil chromatograms, showing: (**a**) the ^1^D GC-FID result indicating the regions 1–5 to be heart-cut; (**b**) the ^2^D GC-FID results of the analysis of all the five heart-cuts with cryofocussing; (**c**) the expanded separation window, comparing the separation of all five selected regions, with retention times given from the introduction of each region to the ^2^D column; and (**d**) the expanded result of heart-cut 4, showing a total retention time of about 24 s. Source: adapted with permission from Dunn et al. [116].

Dunn et al. [104,116] used GC×GC and MDGC to analyse suspected allergens in fragrance products. They found that different column set approaches can be complementary, providing different separations in 2D space. The authors also highlighted the importance of cryogenic focusing to improve peak shapes and separation in the ^2^D column by reducing the effect of peak dispersion (broadening) on the ^1^D column. In another study, the authors applied principles of the comprehensive GC×GC technique to develop a rapid repetitive modulation MDGC method to analyse sequential heart-cuts of peppermint essential oil volatiles, shown in Figure 6. Using a relatively short (~ 6 m long), narrow bore (0.1 mm ID) ^2^D column and a cryogenic modulation system, heart-cuts of 60 s were sampled (Figure 6a) and delivered to the ^2^D column (Figure 6b) to greatly expand separation as shown in Figure 6c with each heart-cut analysis shown from the time of delivery to the ^2^D column; the authors obtained ^2^D separations in 30 s and with up to a 40-fold increase in signal response as illustrated in Figure 6d for the 4th heart-cut.

## 5. Wood 

One of the more interesting notes in the flavour and fragrance industry, woody aroma can be found in, for instance, perfumes and household products to alcoholic beverages due to its storage in wood barrels. Wood is largely composed of biopolymers such as cellulose, hemicellulose and lignin. Smaller amounts of inorganic compounds and extractable substances, such as proteins, amino acids, fatty acids, terpenes, resin, acids, steroids, phenols, aldehydes, ketones, lactones and others are also present; it is in this group that we find the aroma compounds. Although there are many studies investigating the volatile composition of woods, few works concentrate on odour-active components [117,118,119]. 

Tissandié et al. [119] characterised the chemical and odour profile of guaiacwood oil, obtained from the heartwood of *Bulnesia sarmientoi*. GC×GC-FID/MS was applied for identification and quantification of the substances, which was mainly composed of sesquiterpene hydrocarbons and oxides, alcohols, phenols, ethers, aldehydes and ketones. The main odorants were identified by GC-O, highlighting the compounds bulnesol (rosy) and β-ionone (fruity), which had the most odour impact contribution. Typical notes of leather, clove and vanilla were also detected and related to phenolic compounds. 

**Figure 7:**
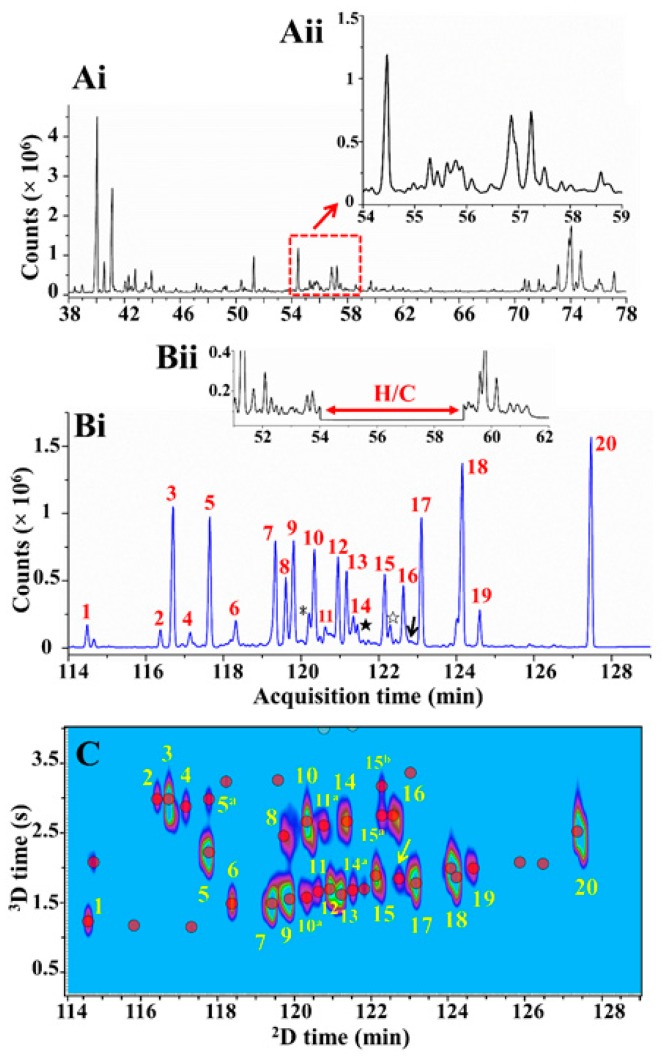
Integrated MDGC-GC×GC analysis of Agarwood (*A. malaccensis*) oxygenated terpenes. GC-FID ^1^D chromatogram (**Ai**) and the expansion of the target region (**Aii**) to be heart-cut. MDGC chromatograms showing the heart-cut (H/C) region in the ^2^D TOFMS detection (**Bi**) and ^1^D FID detection (**Bii**). GC×GC-TOFMS chromatogram (**C**) showing the comprehensive separation of the target region. Source: adapted with permission from Yan et al. [12].

Marriott and co-workers [12,109] assessed the composition of Agarwood (*Aquilaria malaccensis*) oils through different multidimensional approaches. An integrated 3D GC-GC×GC system was used to improve the resolution of oxygenated sesquiterpenes of Agarwood (Figure 7). The conventional 1D analysis is shown in Figure 7A. In the first multidimensional step a 4 min heart-cut was taken using a Deans switch (Figure 7Bii) and cryogenic trap device that gives improved separation (Figure 7 Bi), with the number of detected compounds increased by approximately two-fold compared with 1D GC. However, overlapping peaks were still observed and a subsequent separation was necessary. The application of a cryogenic modulation GC×GC step (Figure 7C) further extended the peak capacity of the heart-cut region by three-fold or more.

Schreiner et al. [117,120] and Ghadiriasli et al. [118], dedicated their studies to the identification of aromas in cedar incense (*Calocedrus decurrens* (Torr.) Florin), Scots pine (*Pinus sylvestris* L.) and oak wood (*Quercus frainetto*), respectively. A MDGC-MS/O system was used to identify trace constituents. In cedar incense, a rot-resistant wood with many commonly used applications, researchers found more than 60 odour substances, with 22 of them being potent odorants, including terpenoids, fatty acid derivatives and phenols as the main classes of odour-active compounds. The pencil-like and carpenter’s shop-like smells were the most dominant impressions in the sample, according to panellists. In Scots pine, 39 odorants were identified, with main classes of terpenoids, aldehydes, ketones and lactones. The highest impacting odour was described as resin-like, attributed to α-pinene, the most abundant compound in all three pine wood samples analysed. In oak wood, a material very frequently used as barrels for alcoholic beverage storage, a total of 91 substances were identified, comprising terpenoids, aldehydes, acids, lactones and phenols. The predominant aroma descriptions were vinegar, green-musty and resin-like.

Natural cork is another woody-related product used as a closure for alcoholic beverage storage and can be a source of off-flavours for the product, mainly related to haloanisoles and attributed to sterilisation [121]. Slabizki et al. [121,122] used MDGC and GC×GC to identify the main cork taint odours, reporting geosmin and 2-methylisoborneol (mouldy and cellar-like), 3-isopropyl-2-methoxypyrazine and 3-isobutyl-2-methoxypyrazine (green), 2,4,6-trichloroanisole (musty-like), and 3,5-dimethyl-2-methoxypyrazine (musty-like) as the main unpleasant odour-active compounds. Furthermore, the compounds 3,4,6-trichloroveratrole and 3,5-dichloroveratrole (musty-like) were described for the first time as cork off-flavours. Multidimensional approaches were indispensable for good separation, identification and quantification. 

## 6. Incense 

**Figure 8:**
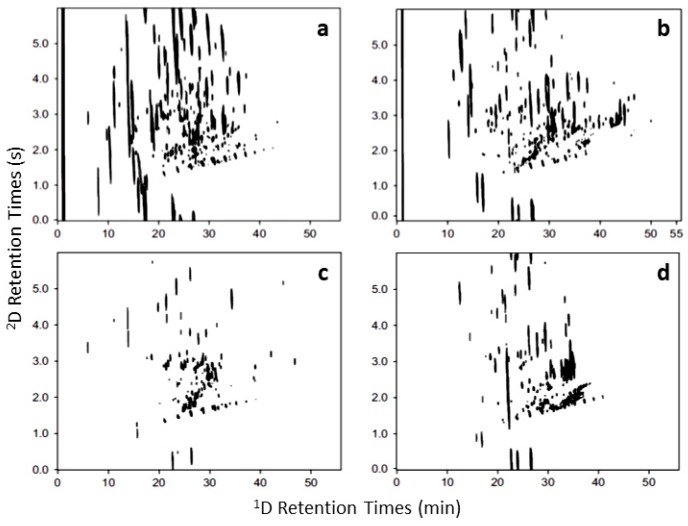
GC×GC-FID volatile profiles from the smoke of four different incense samples: (**a**) lotus-scented, (**b**) red Tibetan, (**c**) medicine herb and (**d**) brown (‘smokeless’). Source: adapted with permission from Tran and Marriott [123,124].

Incenses are aromatisers commonly made with resins and oleoresins, woods, essential oils, flowers, synthetic fragrances, fixatives, etc., which are generally burnt to release a pleasant smell. They are used since antiquity for many purposes, including religious, odour neutralisation and health (aromatherapy) [7,123,124]. Marriott and co-workers [123,124,125] analysed the powder and smoke headspace of four different types of incenses (Figure 8) by GC×GC-FID, GC×GC-TOFMS and GC×GC-ECD/NPD (electron capture detector/nitrogen phosphorus detector) to obtain a qualitative fingerprint and investigate the compounds generated from incense combustion, comparing this with the unburnt incense. The original publication illustrates the relatively few peaks obtained in the incense headspace (mainly aroma compounds) compared with the many new products in the combusted incense. In the first study (2007), the researchers tentatively identified around 120–150 components in the incense powder and more than 200 in the smoke, while in the second study (2008), more than 250 and 1000 compounds were identified in the same samples, respectively. Among the classes present, there was nitrogenous (NPD-active) and halogenated (ECD-active) compounds, *n*-hydrocarbons, terpenoids and phenyl propanoids (essential oils derivatives), fatty acid methyl esters (FAME) and polycyclic aromatic hydrocarbons (PAHs). Different types of nitromusks were also proposed. Naphthalene and biphenyl, both PAH products, were found in all the samples, even the ones claiming to be medicinal incense and environmentally-friendly; they are typical combustion product pollutants. 

Niebler et al. [126] investigated the odour profile of six different Frankincense (*Boswellia sacra* Flueck) samples. A MDGC-MS/O approach was used together with other techniques for better resolution and more reliable compound identification. All samples had very similar profiles with 23 main odorants identified; most were terpenoids. The major compounds and their odour descriptions were *α*-pinene (rosiny, pine), *β*-myrcene (geranium), *p*-cymene (solvent-like, fruity) and limonene (citrus, soapy, fresh). The MDGC technique allowed the authors to separate and evaluate the odour impact of 1,8-cineole and limonene, and also reliably confirm, for the first time, the presence of serratol (also known by cembrenol) in Frankincense samples. The non-terpenoid odour constituents thymoquinone, sotolone, ethyl 3-methylbutanoate and *p*-cresol were also reported for the first time in the samples. 

## 7. Tobacco and Cigarette

Odour-active compounds can be found in some smoking products such as tobacco and cigarettes, arising from the combustion of their components or simply being present in the raw materials. 

Tobacco is a widely studied complex matrix, not only because of the smoking by-products but also because of potential bioactive—e.g., nitrogenous—compounds. The volatile and semi-volatile aroma compounds in tobacco can be divided into three groups: acidic (represented by carboxylic and fatty acids), basic (typically N-containing compounds) and neutral (mainly constituted by free or conjugated aldehydes, ketones, alcohols, alkenes, esters and lactones). They can be present in both the flue-cured tobacco leaves and its smoke and there are some studies applying MDGC and GC×GC to better identify these analytes [127,128,129]. 

Gordon et al. [127] used a MDGC approach to analyse the alkaloid-free essential oil of flue-cured tobacco leaves. The ^1^D separation of about 60 min gave a relatively poor overall precolumn separation. A total of 23 heartcuts—each of about 2–4 min—analysed on the ^2^D column, revealed a series of chromatograms comprising a multitude of peaks, with maybe 1500 compounds observed. The authors identified a total of 360 compounds, of which 80 were reported for the first time in the sample.

Ding et al. [128] developed a method to identify neutral aroma components in tobacco leaves, using a GC×GC-TOFMS system. Wang et al. [129] used the same method to investigate the distribution of 28 neutral aroma components in flue-cured tobacco leaves and classified them into five categories, according to their formation reaction. Ochiai et al. [130] identified and quantified sulfur compounds in smoke extract, many of which are usually present in trace quantities in several matrices though have a high odour impact. Using a MDGC system equipped with a quadrupole time-of-flight MS (qTOFMS) and a sulfur chemiluminescence detector (SCD), they successfully identified 30 compounds that were present at nanogram levels in the smoke. 

Zhou et al. [131] validated a GC×GC-TOFMS method to quantify odour compounds in cigarette smoke, identifying 169 compounds (134 tentatively identified). The main classes contributing to cigarette aroma were alcohols, aldehydes, ketones, acids, pyrroles and pyrazines. Good sensitivity enabled identification of four low-content compounds (*t*-*t*-farnesol, 2-acetyl-5-methylfuran, isoforone and 2,3,5,6-tetramethylpyrazine). 

## 8. Environmental 

Contamination of soil, water and air can lead to the emission of odour substances, which may be hazardous to health. The control of taste and smell of drinking water are important parameters to ensure favourable palatability. Many works focus on detection of odorants in environmental related samples, with GC×GC useful to achieve identification of trace-odour compounds in these samples.

Byliński et al. [132,133] applied GC×GC-TOFMS to identify volatile compounds raised from bio-solid cakes derived from wastewater treatment plants, as well as atmospheric air surrounding this region, which also hosted an oil refinery. They suggested potential odorant markers in the bio-solid cakes based in statistical analysis; 20 compounds were identified with the main classes being alcohols, ketones, sulfur compounds and aromatic hydrocarbons. The sulfur compounds were considered the most determinant to the overall aroma of the sample, due to low olfactory threshold. In the atmospheric air, they found aromatic and aliphatic hydrocarbons, aldehydes, ketones, terpenes and esters as main compounds. The technique allowed the identification, tracking and hazard evaluation of the unpleasant odour pollutants in the area. 

Guo et al. [134,135] and Rong et al. [136], used GC×GC-TOFMS to investigate origins of an unpleasant odour in water from different regions in China. Guo et al. [134,135] indicated four thioether compounds and (*bis*(2-chloroisopropyl) ether) as responsible for swampy/septic odour in the waters from Huangpu and Huai rivers. The authors suggested that 2-methylisoborneol and geosmin are responsible for the musty odour in the first river. Among the 54 substances studied by Rong et al. [136], 2-methylisoborneol was considered the main source of the odour problem in the water from Shiyan reservoir, with its origin mainly attributed to the presence of the cyanobacteria *Pseudanabaena* sp. The GC×GC-TOFMS technique allowed a high sensitivity assessment of compounds from different classes without derivatisation [134].

## 9. Biologics 

Besides the field of plant-derived metabolites (e.g., odorants from essential oils, fruits, vegetables, herbs and spices, flowers, etc.), commented on above, MDGC can be also applied to identify biological-derived odours from humans, animals and insects. 

Cai et al. [137] applied MDGC-MS/O to analyse the ladybug’s (*Harmonia axyridis*) smell in vivo. MDGC improved the separation and evaluation of the main odour impact compounds, related to four pyrazines: 2,5-dimethyl-3-methoxypyrazine, 2-isopropyl-3-methoxypyrazine, 2-*sec*-butyl-3-methoxypyrazine and 2-isobutyl-3-methoxypyrazine.

Saraiva et al. [138] used GC×GC-TOFMS in an ecological study to differentiate three mammal species through the volatiles released by their scats. The technique proved to be useful for wildlife species management as it was possible to distinguish and get information regarding the dietary habits of the studied animals through their scat volatile profiles. 

Following a similar idea, but with different purposes, Ueland et al. [139] used GC×GC-TOFMS to distinguish the horns of different rhinoceros species through odour profiles. Seventeen horn samples (eight white rhinoceros and nine black rhinoceros) were analysed. A consistency in the odour profile of black rhinoceros samples of different origins, clearly differed from the white species samples. The technique proved to be a faster and non-invasive way to identify parts of animals that are illegally trafficked.

Concerning human odours, some studies take advantage of MDGC and GC×GC to explore this field. Buettner et al. [140] used a two-dimensional system to analyse human milk odorants. More than 40 odour-active compounds were identified and can be used to study the impact of diet and lifestyle habits of women based on their milk composition. Kuhn et al. [141] assessed the body odours of monozygotic twins by GC×GC-TOFMS. Important carboxylic acids related to the sweat odour, allowed the proposition of a possible contribution of genetics to human body scent, as the twins’ samples showed greater similarity in profiles than those from unrelated individuals. Doležal et al. [142] evaluated the scent of women’s skin, focusing on the less volatile compounds by using GC×GC-TOFMS. The study resulted in separation and identification of more than 137 substances, including fatty acids and their esters, ketones, aldehydes, esters and alcohols.

In textile science, GC×GC-TOFMS was applied to obtain a profile and chemometric study of human axillary sweat in two different knit fabrics. The results showed useful application to distinguish different types and wash treatment of fabric, as well as identify the gender of the wearer. Carboxylic acids, aldehydes, ketones, and alcohols were the classes selected, through application of chemometric algorithms, to distinguish between cotton and polyester textiles. Moreover, aldehydes, alcohols and some aromatics were the most representative to discriminate the sex of the user [143].

## 10. Forensics

Forensic science comprises a wide variety of analysis objectives, such as human scent and remains, criminal environmental pollution, arson and explosives, illicit drugs, etc. [3,144]. Several studies exploring the potential of GC×GC and MDGC techniques in this field have been published, as reviewed by Gruber et al. [144], although some limitations such as method standardisation, consistency and interpretation of results are still faced. 

Fingerprinting of volatiles released by the human body (dead or alive) can be used for location purposes. This issue is especially interesting to aid the training of detection dogs, which are usually exposed to human scents to track pieces of evidence in crime scenes or rescue missions [144,145]. Studies have been conducted with different types of odour samples, such as human hands [146,147], blood [145,148,149], decomposition odour chemical mixtures [150], decomposition soil and adipocere from the death scene [151], human and animal cadavers [152,153,154,155,156,157,158,159,160,161], etc. GC×GC-TOFMS analysis was the most applied technique and low/mid polarity stationary phases preferred as ^1^D, while for ^2^D, the stationary phase is commonly polyethyleneglycol (wax).

Focant and coworkers applied GC×GC-TOFMS to analyse odour volatiles for forensic applications, covering cadavers, blood, environmental pollutants, chemical odour products and explosives [149,154,155,156,157,158,159,160,161,162,163,164]. They evaluated the composition of some commercial chemical mixtures of odorants used in the training of cadaveric detection dogs, to mimic the human decomposition smell. The authors reported seven compounds present in products that are not related to cadaveric decomposition, indicating that these may not be suitable for the stated purpose [150]. 

The same research group analysed pig carcasses as human analogue cadavers; samples of soil and adipocere collected from the death scene or around pig carcasses, human remains and gas reservoirs inside a cadaver [151,152,153,154,155,157,158,159,160,161]. Many chemical classes are present among the decomposition profiles: alcohols, carboxylic acids, aromatics and sulfides were the main classes of substances identified by Stadler et al. [153]; some potential key decomposition compounds were 1-butanol, 1-octen-3-ol, 2-and 3-methyl butanoic acid, hexanoic acid, octanal, indole, phenol, benzaldehyde, dimethyl disulfide and trisulfide. Stefanuto et al. [160,161] compared the odour volatile profiles of human and pig carcasses and also observed that sulfides may be important markers to discriminate the decomposition volatiles from different organs in human intracadaveric gas reservoirs. Dubois et al. [151] analysed the soil and adipocere samples collected from the decomposition site of a real crime scene, seven days after body removal. The GC×GC-TOFMS profile results showed advantages, such as detection of trace compounds, high selectivity, accuracy and high resolution. The image offers a simple way to locate analytes through their compound classes and to recognise separation patterns, enabling an easier differentiation of samples collected from different places and facile communication of scientific evidence. 

Stefanuto et al. [164] studied the headspace of taggants and other minor volatile compounds associated with commercial thermally-unstable explosives: 1,3,5-trinitroperhydro-1,3,5-triazine (RDX); [3-nitrooxy-2,2-bis(nitrooxymethyl) propyl] nitrate (PETN) and 2-methyl-1,3,5-trinitrobenzene (TNT). GC×GC-TOFMS analysis revealed the presence of plasticisers (phthalates) and stabilisers (amino benzoic compounds), as well as nitro- and nitroso- compounds that can be from the explosive or its degradation products. Obtaining a fingerprint signature for each product, with respect to specific characteristics of the manufacture and storage conditions, can be useful to aid the training of detection dogs, assist tracking of the explosive’s origin and to provide faster confirmatory identification of explosives by the forensic laboratory.

**Figure 9:**
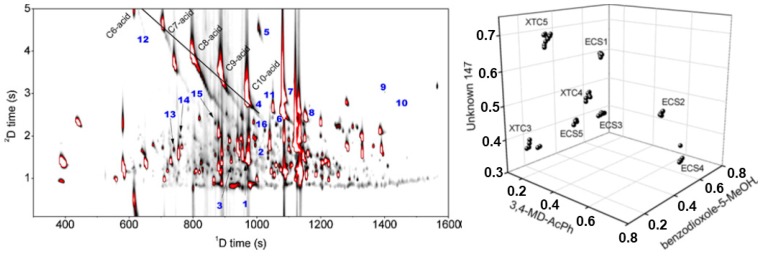
GC×GC-TOFMS volatile profile of an ecstasy sample, along with the location of the 16 compounds selected for the profile correlation and the identified fatty acids (**left**). 3D plot of the linear discriminant analysis (LDA) analysis with three selected compounds, showing the clear clustering of the eight groups of ecstasy samples (**right**). Source: adapted with permission from Mitrevski et al. [165].

The determination of key volatile compounds of illicit drugs was the focus of a number of studies [165,166,167]. Mitrevski et al. [165] used GC×GC-TOFMS to assess the volatile composition of ecstasy samples, from different countries, seizures and tablet physical characteristics (logo, colour, diameter and thickness), and developed a method to determine their origin through the profiles of some specific organic impurities. Figure 9 shows a typical GC×GC 2D result for ecstasy and which contains clear signatures of the processing of the sample from the safrole starting material, since it was possible to identify reaction intermediates in the final product. Enhanced efficiency and sensitivity were achieved with the comprehensive approach, which enabled the clear discrimination of the samples into eight groups in the linear discriminant analysis (LDA) plot. Three compounds were chosen to discriminate the samples: 3,4-MD-acetophenone, 1,3-benzodioxole-5-MeOH and unknown 147. Macedonia samples (named XTC) were rich in *N*-formyl-MDMA and *N*-acetyl-MDMA, while Australia samples (named ECS) have more 3,4-methylenedioxypropane and 3,4-methylenedioxyacetophenone. 

Rice et al. [167] developed a MDGC-MS/O method to compare odours emitted from marijuana, cocaine and heroin samples with synthetic pseudo-narcotic scent formulations. The authors reported that the most abundant compounds in headspace of the real samples were not of the greatest odour impact. Furthermore, the pseudo-scent formulations analysed did not match the chemical concentration and odour impact of the analytes from real samples. The marijuana-like product had more alcohols and aromatics; the cocaine-like product had more alcohols, aldehydes, aromatics, esters, ethers, halogenates, hydrocarbons, ketones and N-containing compounds; and the heroin-like product contained many more acids, alcohols, aromatics, esters, ketones, and N-containing compounds than the real samples. The use of MDGC-MS/O was indispensable to achieve a well-resolved separation, detect minor compounds with high odour impact and achieve simultaneous aroma evaluation using the two detectors.

## 11. Plastic Products Applications

Buettner and co-workers [168,169,170,171] used MDGC-MS/O techniques to investigate intense malodours and hazardous chemicals in children toys, balloons, balls, hairbrushes, inflatable pillows, elastic therapeutic tapes, artists’ acrylic paint and other commercial products. Some of the most intense and unpleasant odours detected in these products were classified as car tyre/rubber-like, pungent, burning sensation, artificial leather-like, plastic-like, glue-like and nail-polish-like. The most frequent compound classes identified were aldehydes, carboxylic acids, lactones, indoles, phenols, alcohols, hydrocarbons and PAHs. 

## 12. Conclusions and Future Perspectives

The multi-separation-column techniques of MDGC and GC×GC are continually being improved, and find an increasing applications base as researchers explore a diverse portfolio of investigations. For convenience, the above applications are gathered into a single table (Table 2) that indicates column sets used for the variety of studies of different matrices. 

Not surprisingly, many different matrices related to aroma and flavour samples and their chemical composition are fertile areas for these techniques, primarily due to the many compounds of similar structures in the complex suite of analytes. This means compound overlap is very common in samples when using a ^1^D GC separation. MDGC and GC×GC address these shortcomings. Since minor compound abundances are often of interest, this also demands improved separation. These techniques make possible, not only the qualitative and quantitative assessment of the analytes, but also GC×GC offers a simpler approach to fingerprint pattern recognition since it may be based on a 2D ‘picture’ representation, which is useful to distinguish the type, origin, variety and other intrinsic characteristics of the samples. Moreover, discovering new odorants, as well as enantiomeric separation and enantiomer ratio determination are supported by these methodologies. 

Beyond the applications already explored, there is further space for innovation, and it is now accepted that multidimensional analysis approaches can offer such a wide range of valuable and conspicuous information for olfactometry, mass spectrometry and general characterisation. Emerging applications of these techniques can be as an auxiliary in the investigation and control of terrorist attacks, by means of understanding and improving the training of detection dogs, as well as the identification and tracking of explosives and toxic gases. 

Another potential application is in the clinical diagnostics of diseases through their odour signatures and biomarkers. There are some technological e-nose devices that have already been studied for this purpose, which could help in the detection of a range of illnesses, from pneumonia to lung cancer, in a fast, non-invasive and inexpensive way, as discussed by Friedrich [172]. The use of new detectors, such as vacuum ultraviolet (VUV) and barrier discharge ionisation (BID) and post-column reaction with FID, coupled to gas chromatography techniques await evaluation for their role on improving identification for flavours and aromas. Similarly, newer commercial phases such as ionic liquids, have been little used in flavour analysis for MDGC and GC×GC, and limited in general 1D analysis of these samples, so cannot be fully evaluated here. Improved multidimensional methods are also important in the identification and quantification of allergens, skin sensitisers and toxic compounds present in trace amounts in fragranced products, as well as the study of their interaction with the body. 

With the expanding demand for sustainable products and processes, the applications of MDGC and GC×GC, especially with determination of enantiomeric and stable isotope ratios, could be also extended to environmental and ecological monitoring, processing and quality control of products or chemical characterisation in matrices not yet deeply explored.

## Figures and Tables

**Table 1:** Multidimensional (MDGC) and comprehensive two-dimensional gas chromatography (GC×GC) general features and counterpoints.

Technique	Features	Pros	Cons
MDGC	- Target analysis (heart-cut; H/C) - Target peaks/regions are transferred according to retention times in ^1^D, and analysed in ^2^D - ^2^D column usually has similar dimensions to the ^1^D - A cryotrap can be used to focus the transferred analyte peaks/regions	- Allows enrichment of target analytes or fractions through multiple injections - Provides increased resolution on the ^2^D column and can improve detection specificity and sensitivity - Stereoisomer resolution uses a chiral ^2^D column and improves resolution from interfering peaks	- Requires a cryogenic trap to reduce ^1^D dispersion - Requires a switching valve for on-line H/C programming - Not designed to resolve the full sample; - Requires extra GC program to elute the ^2^D column, unless on-the-fly operation is used
GC×GC	- Non-target analysis; applied to all sample compounds - Modulator sub-samples peaks as small slices to ^2^D, giving a 2D plot - ^2^D ideally separates overlapped ^1^D peaks - ^2^D column is shorter, to separate transferred compounds before the next modulation	- Full 2D sample resolution can be achieved - Stereoisomer resolution uses a ^1^D chiral column - The 2D image generated provides excellent profiling/differentiation of samples. - Cryogenic modulation leads to response increase	- Requires a modulator; some can be costly - Method set-up can be more complex - Software and interpretation can be more convoluted - Personnel need special training

**Table 2:** Summary of the applications of MDGC and GC×GC in aroma analysis, referred to in this article.

Samples/Column Sets *	References
**Food**	
***Fruits and nuts****:* orange juice; *Citrus* spp.; pineapple; passion fruit; strawberry; raspberry; blue honeysuckle; chokeberry; bilberry; cocoa; hazelnuts.	[21,22,23,24,25,26,27,28,29,30,31,32,33,34,35,36,37]
Column sets: HP-FFAP/DB-5; Rtx-5MS/beta-DEXTM 55; Equity-1/SolGel-Wax	
***Honey***	
Column set: ZB5-MS/BPX50	[38]
***Dairy products****:* butter; dairy cream	
Columns sets: BP21/BPX35	[39,40]
***Flour and pasta***	
Columns sets: SLB-5MS/Mega-Dex Det	[41]
***Meats and Seafood****:* beef; lamb; ham; shrimp; seabass; eel	
Columns sets: DB-1/DB-225; HP-5 MS/BPX50; Rtx-5MS/DB-17	[42,43,44,45,46,47,48,49]
***Seasonings and related samples****:* coriander; curry leaves; fennel seeds; ginger; virgin olive oil; rapeseed oil; vinegar; tomato-onion puree; truffles.	
Columns sets: BPX5/BP20; HP-InnoWax/DB-1; Rxi-5Sil MS/BPX50; DB-WAX/DB-5	[50,51,52,53,54,55,56,57,58,59,60,61,62]
**Drinks and Beverages**	
***Non-alcoholic drinks****:* teas; coffee	[9,63,64,65,66,67,68,69,70,71,72,73]
Columns sets: Rxi-5Sil MS/Rxi-17; DB-5/Supelcowax; DB-Wax/Cyclosil B; TC-WAX/BETA DEX 225; DB-FFAP/DB-5	
***Alcoholic beverages****:* wine; brandy; beer and hop; cider; vodka; whiskey; cachaça; spirits	
Columns sets: DB-FFAP/DB-5; DB-5/DB-225; DB-WAX/DB-5; DB-1/DB-WAX	[8,9,14,15,68,69,74,75,76,77,78,79,80,81,82,83,84,85,86,87,88,89]
**Essential oils (E.O.) and fragrances**	
flower odorants; vetiver E.O. and vetiveryl acetates (chemical and enzymatic products); rosewood leaves E.O.; eucalyptus E.O.; lavender E.O.; tea tree E.O.; peppermint E.O.; perfumes; allergens	[18,20,25,52,91,92,93,94,95,96,97,98,99,100,101,102,103,104,105,106,107,108,109,110,111,112,113,114,115,116]
Columns sets: VF-5MS/DB-wax; DB-WAX/DB-5; Supelcowax/IL-59/BPX5	
**Wood**	
guaiacwood; agarwood; oak wood; scots pine; cedar; cork	[12,109,117,118,119,120,121,122]
Columns sets: Supelcowax/Rxi-5Sil MS; VF-5MS/DB-wax; DB-FFAP/DB-5	
**Incenses**	
lotus-scented; red Tibetan; medicine herb; brown (smokeless); frankincense	[123,124,125,126]
Columns sets: BPX5/BP20; DB-FFAP/Rxi-5	
**Tobacco and Cigarette**	
tobacco leaves, tobacco smoke, cigarette smoke	[127,128,129,130,131]
Columns set: HP-5MS/DB-17 HT; DB-Wax/DB-1701; Rxi-5MS/Rxi-17	
**Environmental**	
biosolid cakes; atmospheric air; water	[132,133,134,135,136]
Columns sets: Equity 1/SolGel-Wax; Rxi-5Silv/Rxi-17	
**Biologics**	
ladybugs; mammal scats; rhinoceros horns; human milk; human sweat/skin odour	[137,138,139,140,141,142,143]
Columns sets: Rtx-5MS/DB-Wax; Rxi-624sil MS/Stabilwax	
**Forensics**	
pig and human cadavers; human remains; human hands scent; blood; environmental pollutants;	[144,145,146,147,148,149,150,151,152,153,154,155,156,157,158,159,160,161,162,163,164,165,166,167]
explosives; dog training mimetic odours; illicit drugs	
Columns sets: Rxi-624Sil MS/Stabilwax; Rxi-5Sil MS/Rxi-17Sil MS	
**Plastic products**	
children toys; balloons; balls; hairbrushes; inflatable pillows; elastic therapeutic tapes; acrylic paint	[168,169,170,171]
Columns set: DB-FFAP/DB-5	

* Common column sets used in the literature (equivalent columns or different column sets were also observed).
